# Aggressive Acute Marjolin's Ulcer Arising in a Burn Scar

**DOI:** 10.1155/2022/8329050

**Published:** 2022-11-08

**Authors:** Adela Alvarez Rio, Josep Oriol Roca Mas, Daniel Navarro Sanchez, Ivan Monge Castresana, Gonzalo Joaquin Soroa Moreno, Jaime Estrada Cuxart

**Affiliations:** Plastic & Reconstructive Surgery, Hospital Universitario Son Espases, Palma de Mallorca, Spain

## Abstract

Marjolin's ulcer is one of the clinical variants of squamous-cell carcinoma. It is a highly aggressive disease that develops from chronic wounds. Almost 65% of these lesions have been diagnosed on underlying burn scars. Although the mean latency time between the primary lesion and the apparition of the ulcer is around 25 years, some cases with an early debut have been described. Squamous-cell carcinomas arising in chronic wounds are typically aggressive and are related with a poor prognosis due to their late diagnosis. Therefore, it is important to recognize symptoms that indicate malignant degeneration of chronic wounds, allowing the clinician to make an early diagnosis in order not to delay the surgical treatment that is required to improve the global survival of the patient. The time elapsed between our patient's burn and the appearance of Marjolin's ulcer was only 7 months, drawing attention to its fast and aggressive progression.

## 1. Introduction

Squamous-cell carcinoma is a malignant tumour developed from epidermal keratinocytes. A skin biopsy is always required to confirm the diagnosis. Among its clinical variants, we can find Marjolin's ulcer.

Marjolin's ulcer, described for the first time in 1963 by French surgeon Jean-Nicholas Marjolin [1], is considered a highly aggressive disease that develops from chronic wounds and skin scars. Almost 65% of these ulcers have been diagnosed on underlying burn scars, raising the malignancy degeneration rate of a burn to 1.5% [2].

Although other entities have been related with Marjolin's ulcer, the most frequently described histology has always been squamous-cell carcinoma, with rates around 71% [3].

Malignant degeneration is usually slow. Albeit the mean latency time between the primary lesion and the onset of the ulcer is around 25 years, some cases with an exceptionally early debut have been described, with a latency time of around 2 years [2]. Marjolin's ulcers can therefore be described as acute if they appear in the first 12 months since the primary lesion or as chronic if they appear in the posterior 12 months, the latter being the majority [3]. All of these facts indicate that the earlier the primary lesion appears, the more the likelihood of developing this pathology over time.

Squamous-cell carcinomas developed on chronic wounds are typically aggressive and are related with a poor prognosis due to the confusion with a classic wound and the delay in its diagnosis [4].

In our case report, the latency time between our patient's burn and the appearance of Marjolin's ulcer was of only 7 months, drawing attention to its fast and aggressive progression, despite the usually slow development of this disease, as previously described.

## 2. Case Report

A 48-year-old male patient, on chronic opioid treatment for multiple disc hernias, consulted the emergency room about an extensive 7-month-evolution thermic burn in the dorsum that has presented a torpid evolution.

**Figure 1:**
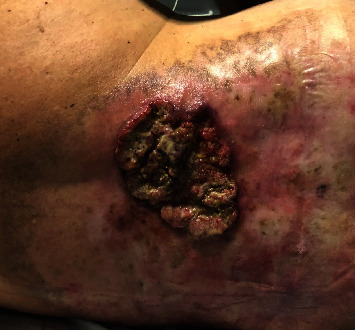
Exophytic ulcer 7-months after a thermic burn in the dorsal region. First patient visit to emergency department.

He presents a large, exophytic, malodorous ulcerated lesion on the back with an approximate length of 15 × 10 cm. In the physical exploration, there are palpable lymph nodes in the left groin, without other alteration. The patient refers to a dermal burn 7 months ago that has worsened gradually despite multiple antibiotic treatments and local dressings with all kinds of antiseptics and bactericides in his primary care centre given by different medical teams (Figure 1). He had never been sent to a specialised centre despite the bad local evolution.

During his stay in the emergency room, two biopsies of the lesion were taken. The histological results are described as moderately differentiated infiltrative squamous-cell carcinoma that invades deep reticular dermis.

Given the results of the biopsy, the case is exposed to the skin cancer committee, and a PET-CT scan is prescribed to complete the case study and get information about the spread of the disease. Results show pathological activity in the left groin and doubtful activity in the right axilla. A fine-needle puncture-aspiration assessment is performed on the right axilla, with negative results for malignancy.

The patient underwent surgery for the first time. We performed a wide resection of the primary tumour with 2 cm lateral margins and including deep lumbar fascia into the resection. A regulated left inguinal lymph node dissection was performed in the same surgery. The lumbar defect was reconstructed with meshed partial-thickness skin grafts.

**Figure 2:**
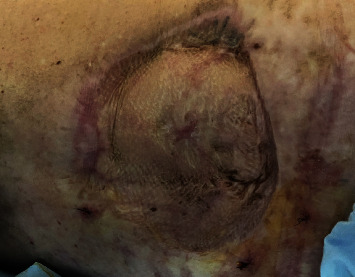
Dorsal region after wide surgical extirpation and reconstruction with meshed split-thickness skin grafts.

Histological results evidenced a moderately differentiated infiltrative squamous-cell carcinoma with free surgical margins and a metastatic affectation in 6 out of the 14 lymph nodes extirpated. Postsurgery evolution was favourable, with the results after a 6-month follow-up shown in Figure 2.

**Figure 3:**
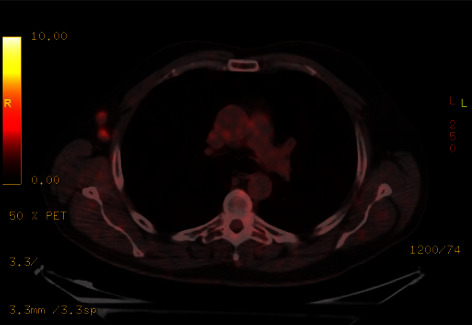
PET-CT scan 6 months after first diagnosis showing two metastasic lymph nodes in the right axilla.

7 months after diagnosis, during a routine check-up, 2 axillary lymph nodes become palpable. A new PET-CT scan is performed, and the progression of the disease is acknowledged as the images are positive for malignancy in that axilla (Figure 3).

A second surgery is performed two weeks later. A regulated right axillary lymph node dissection is accomplished. Results inform metastatic affectation in 7 out of 31 lymph nodes extirpated. Due to the aggressiveness of the disease, radiotherapy was elected as an adjuvant treatment for this patient.

6 months after the last surgery and as far as our follow-up has arrived, the patient still continues to be stable without any further progression of the disease either locally or systemically.

## 3. Discussion

Malignant degeneration of a burn in the first 12 months since the acute lesion is something unusual. A review of the literature shows that the latency period between initial burn and subsequent malignancy can vary from 20 to 35 years, as shown in most series [5–7]. Xu et al. reported two Marjolin Ulcers arising over chronic foot wounds within 1 and 2 years, and Love RL presented one Marjolin's ulcer arising over a burn scar [8, 9]. There are very few cases with a very low number of patients reported in the literature about acute Marjolin's ulcers with such a fast development, even less about this disease over a recent burn scar, so describing new cases of this entity is important as it tends to be infradiagnosed and therefore, infratreated [8–10].

In the majority of described cases of acute Marjolin's ulcer, the most frequently isolated histological variant has been basal-cell carcinoma, leading to a more favourable prognosis [11].

Even though Marjolin's ulcer pathogenesis is uncertain, its multifactorial origin is clear, and it is known that genetics and ambience can influence its development. Zones subjected to an acute lesion, such as the bed of a burn, suffer from constant inflammation, which could promote the activation of tumour cells that were already there and trigger DNA mutations [12]. Furthermore, an obliteration of the lymphatic vessels and a decrease in the blood flow accompany the primary lesion, limiting the local tissue's immunological ability to defend from aggressions.

These ulcers tend to be treated on an outpatient basis by different health care professionals who are usually untrained, are inexperienced, and lack diagnostic weapons with this kind of lesions. All of this leads to a delay in this pathology's diagnosis and treatment.

Consequently, all of this translates into a poor prognosis for this disease with a 21% global mortality rate [13]. Undoubtedly, histologic grade of the tumour and lymph node affectation in the moment of diagnosis are the most important prognosis factors. 3 years after diagnosis, the global survival of patients with Marjolin's ulcer is from 65 to 75%, numbers that descend to 35–50% if there is metastasis from the beginning [13].

Hence, it is very important to recognize signs and symptoms that denote a possible malignant degeneration in chronic wounds and burns so an early biopsy can be taken in order to establish the diagnosis as soon as possible.

Despite the fact that developing a Marjolin's ulcer a few months after a primary burn is not very common, if the ulceration is maintained chronically for longer than 3 months and malignancy indicators appear (such as bleeding, excessive pain, or severe exophytic granulation tissue), any clinician must take into account this diagnose and do not delay the biopsy of the lesion.

If a squamous-cell carcinoma is confirmed, due to its high aggressiveness, it is mandatory to start treatment as soon as possible with a radical extirpation with wide margins. An early staging has to be performed, and a regulated lymph node dissection or sentinel lymph node biopsy should be performed if required. Adjuvant chemotherapy and radiotherapy can be added if the case requires it [14].

## 4. Conclusion

A biopsy of any suspicious lesion should not be delayed even though little time has elapsed since the original burn. Alarm signs and symptoms must be searched for if the evolution of a wound, ulcer, or burn is torpid and more than 3 months have passed. A biopsy should be taken without delay in order to confirm the diagnosis and start medical treatment.

An ample surgical removal must be performed, accompanied by lymph node dissection or sentinel lymph node biopsy when required. After the surgery, every patient should be closely followed up to watch over possible relapses or metastases that can appear.

We want to emphasise in this report the importance of this forgotten entity and remark that, even when the onset is acute, it should be taken into account due to its aggressiveness and fatal consequences if the diagnosis is delayed.

## References

[1] Cruickshank A. H., McConnell E. M., Miller D. G. (1963). Malignancy in scars, chronic ulcers, and sinuses. *Journal of Clinical Pathology*.

[2] Pekarek B., Buck S., Osher L. (2011). A comprehensive review on Marjolin’s ulcers: diagnosis and treatment. *The Journal of the American College of Certified Wound Specialists*.

[3] Tobin C., Sanger J. R. (2014). Marjolin’s ulcers: a case series and literature review. *Wounds*.

[4] Al-Zacko S. M. (2013). Malignancy in chronic burn scar: a 20 year experience in Mosul-Iraq. *Burns*.

[5] Iqbal F. M., Sinha Y., Jaffe W. (2015). Marjolin’s ulcer: a rare entity with a call for early diagnosis. *BMJ Case Reports*.

[6] Choa R., Rayatt S., Mahtani K. (2015). Marjolin’s ulcer. *BMJ*.

[7] Kerr-Valentic M. A., Samimi K., Rohlen B. H., Agarwal J. P., Rockwell W. B. (2009). Marjolin’s ulcer: modern analysis of an ancient problem. *Plastic and Reconstructive Surgery*.

[8] Xu S., Kang Y., Soeharno H., Yeo N. (2020). Acute Marjolin’s ulcer in chronic foot wounds with previous negative biopsy: report of two cases. *Malaysian Orthopaedic Journal*.

[9] Hammond J. S., Thomsen S., Ward C. G. (1987). Scar carcinoma arising acutely in a skin graft donor site. *The Journal of Trauma, Injury, Infection, and Critical Care*.

[10] Love R. L., Breidahl A. F. (2000). Acute squamous cell carcinoma arising within a recent burn scar in a 14-year-old boy. *Plastic and Reconstructive Surgery*.

[11] Gaughan L. J., Bergeron J. R., Mullins J. F. (1969). Giant basal cell epithelioma developing in acute burn site. *Archives of Dermatology*.

[12] Fleming M. D., Hunt J. L., Purdue G. F., Sandstad J. (1990). Marjloin’s ulcer: a review and reevaluation of a difficult problem. *Journal of Burn Care & Rehabilitation*.

[13] Saaiq M., Ashraf B. (2014). Marjolin’s ulcers in the post-burned lesions and scars. *World Journal of Clinical Cases*.

[14] Aydogdu E., Yildirim S., Aköz T. (2005). Is surgery an effective and adequate treatment in advanced Marjolin’s ulcer?. *Burns*.

